# Community Awareness and Health Risk of Heavy Metals Through Consumption of Sardine (*Rastrineobola argentea*) From Lake Victoria, Tanzania

**DOI:** 10.1002/fsn3.70898

**Published:** 2025-09-03

**Authors:** Happiness Kemilembe Venant, Dickson W. Lwetoijera, Neema Kassim

**Affiliations:** ^1^ Ifakara Health Institute (IHI) Dar es Salaam Tanzania; ^2^ The Nelson Mandela African Institution of Science and Technology (NM‐AIST) School of Life Sciences and Bio‐Engineering (LiSBE) Arusha Tanzania

**Keywords:** community awareness, heavy metal, human health risk, Lake Victoria, sardine, Tanzania

## Abstract

Sardine ( *Rastrineobola argentea* ) is a crucial food source for East African communities around Lake Victoria, providing essential nutrients such as protein, amino acids, minerals, and vitamins. However, heavy metal contamination in aquatic environments threatens its safety and may affect human health. This study assessed community awareness of heavy metal contamination and estimated the health risks from sardine consumption in Lake Victoria, Tanzania. A cross‐sectional study was conducted in Mwanza, Kagera, and Mara regions. A semi‐structured questionnaire assessed awareness and consumption, while a Flame Atomic Absorption Spectrometer quantified cadmium and lead concentrations in sardine samples. About 80% of 425 participants were women, and the majority (96.2%) were unaware of heavy metal contamination. Median concentrations in sardines were 0.081 ± 0.057 mg/kg (cadmium) and 0.012 ± 0.005 mg/kg (lead). Estimated daily intakes in mg/kg/day were cadmium (1.043–1.175 × 10^−5^) and lead (2.704–3.047 × 10^−5^). The target hazard quotients were 0.014 (cadmium) and 0.024 (lead), with hazard indices ranging from 0.0053 to 0.0060. Target cancer risk values ranged from 1.03 × 10^−9^ to 1.70 × 10^−5^. Heavy metal levels in sardines and daily exposure were within permissible limits set by national and international standards. Target hazard quotients, hazard indices, and cancer risk values were all below tolerable limits, indicating no significant health risks from consuming sardines from Lake Victoria. These findings suggest that although community awareness of the risk of heavy metal remains low, the concentrations of cadmium and lead in sardines and the associated dietary exposure do not pose significant health risks to consumers. The study recommends continuous monitoring, awareness campaigns targeting stakeholders, and promoting raised racks for drying to reduce contamination. These findings offer preliminary evidence to inform decisions by environmental, food, and fish authorities, local governments, and fishing communities, and raise awareness among traders and consumers.

## Graphical abstract

Sardine (*Rastrineobola argentea*) is a crucial food source for East African communities around Lake Victoria. Estimated daily intake, hazard quotients, and cancer risk values for cadmium and lead indicated no significant health risks. Sardine from Lake Victoria was deemed safe for human consumption.

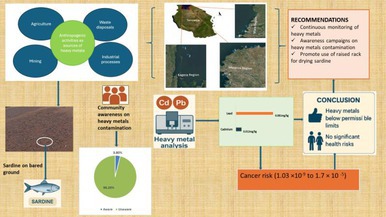

## 1 Introduction

Sardine ( *Rastrineobola argentea* ) is an essential source of food in the East African countries around Lake Victoria (LVFO 2012). It contains essential nutrients such as digestible protein, essential amino acids, minerals, and vitamins (Abdulkarim et al. 2015). Sardine from Lake Victoria contains about 53% to 59% w/w of protein (Kabahenda et al. 2011). It also contains essential lipids with fatty acids such as omega‐3 (0.473 g) and omega‐6 (0.509 g) (Santos et al. 2023), that are known to lower plasma triglycerides, blood pressure, resting heart rates, as well as reduce inflammation, and increase myocardial filling and efficiency, and brain development (Olgunoglu 2017). Sardines contribute about 60% of the total fish catch in the lake and are a main source of livelihood for the people. Dried sardines are commonly consumed by the local people around the lake and are sold at domestic and regional markets, such as Burundi, Rwanda, the DRC, Zambia, Malawi, and South Sudan (LVFO 2012). However, fish including sardines trading and consumption are threatened by extensive contamination of aquatic environments including heavy metals, which in turn affect human health (Duan et al. 2022).

Mining and industrial activities, including metal smelting, recycling, and fertilizer use, are potential sources of cadmium and lead around Lake Victoria, particularly in Mwanza, Kagera, Mara, and Kahama (Hongo and Mjema 2002). Mwanza also has multiple factories, including fish processing, textile, and chemical plants, which release wastes into the lake, contributing to lead pollution from various products such as pigments, steel, and ceramic products, fuel, batteries, cables, food packaging, and glassware (Ob and Lo 2019).

Heavy metals enter Lake Victoria mainly through river inflow from rivers such as Magogo, Moene, Nyashishi, Kagera, Biharamulo, Simiyu, Gorometi, and Mbalageti. Additional contamination stems from runoff associated with small‐scale mining, gold processing, sewage, and industrial waste, posing significant risks to aquatic plants, animals, and surrounding human populations (Baby et al. 2010). Globally, heavy metals are recognized as major pollutants in aquatic ecosystems, predominantly originating from human activities such as wastewater discharge, improper disposal of hazardous waste, and agricultural inputs. In contrast, natural sources such as erosion or weathering of parent rock materials contribute relatively little to the overall metal load (Rakib et al. 2022; Saravanan et al. 2024).

When these metals are present in soil and water, they can be taken up by plants, animals, and other organisms, leading to accumulation (Kumari and Mishra 2021). Ingesting food or water contaminated with heavy metals leads to exposure (WHO 2023). Lead accumulates in bones and can be released into the bloodstream during pregnancy, affecting the fetus. Heavy metals such as cadmium (Cd) and lead (Pb) present significant public health risks due to their toxicity, even at low concentrations. Cadmium accumulation can cause renal failure, while lead exposure can lead to miscarriages, increase the risk of kidney damage and high blood pressure, and impact the central nervous system, brain, and liver, potentially causing convulsions and coma (WHO 1972; FAO/WHO 1972).

To date, there is limited information on heavy metal contamination of the lake. Studies conducted on the Kenyan side of Lake Victoria indicated that fish were contaminated above the FAO/WHO limit of 0.3 mg/kg (FAO/WHO 1972), with Cd (0.48 ± 0.013 to 3.00 ± 0.009 mg/kg) and Pb (3.42 ± 0.045 to 12.78 ± 0.108 mg/kg) in fish species including sardines from Nakuru Market (Esilaba et al. 2020) and Pb (0.57 mg/kg) in dried *Rastrineobola argentea in the* Karungu site, Kenya (Oyoo‐Okoth et al. 2010). In Uganda, a study reported a Cd concentration of 0.57 μg/g in sun‐dried *Rastrineobola argentea* collected from 10 landing sites in Lake Victoria (Mbabazi and Wasswa 2010). The study concluded that excessive consumption of sardines could pose a health risk, with the contamination likely resulting from untreated industrial discharges and other anthropogenic effluents entering Lake Victoria. In Tanzania, sediment samples from rivers and streams entering the lake via the southern zone of Mwanza municipality have revealed high levels of heavy metal contamination, in which lead (Pb) concentrations were found to be 345 ± 93 mg/kg, exceeding the limit of 200 mg/kg set by the National Environment Management Council (NEMC) of Tanzania (NEMC 2007). Kishe and Machiwa (2003) reported elevated concentrations of lead (54.6 ± 11.1 mg/kg) in sediments along the urban shoreline of Mwanza, likely due to industrial and urban runoff. The same study recorded the highest cadmium (Cd) levels (7.0 ± 2.1 mg/kg) near river mouths, indicating riverine discharge as a major source. Similarly, Machiwa (2004) reported elevated concentrations of Pb (58.1 ± 17.6 mg/kg) and Cd (7.0 ± 2.1 mg/kg) in sediments at sites adjacent to the Mirongo river mouth. These findings underscore the importance of geographical factors, including proximity to industrial zones and river mouths, in influencing metal accumulation in sediments. While efforts have been directed at big fish such as Nile perch, which accounts for 59.3% of fish export from Lake Victoria, there is a critical lack of information on the safety of sardines. Studies specifically quantifying heavy metals in sardines ( *Rastrineobola argentea* ) are limited. Only one study reported a Pb concentration of 0.01 mg/kg in sardine samples collected from offshore processors in Mwanza, indicating low contamination levels within acceptable limits for human consumption. Previous studies have investigated the presence of bacteriological contamination in *Rastrineobola argentea* from Lake Victoria, Tanzania (Baniga et al. 2017). While other studies have investigated heavy metals and pesticides in Nile perch and tilapia (Wenaty et al. 2019; Mhina 2016), this study therefore aimed to assess community awareness of heavy metal contamination and the health risk of lead and cadmium through the consumption of sardines from Lake Victoria, Tanzania.

## 2 Materials and Methods

### 2.1 Study Area and Design

**FIGURE 1:**
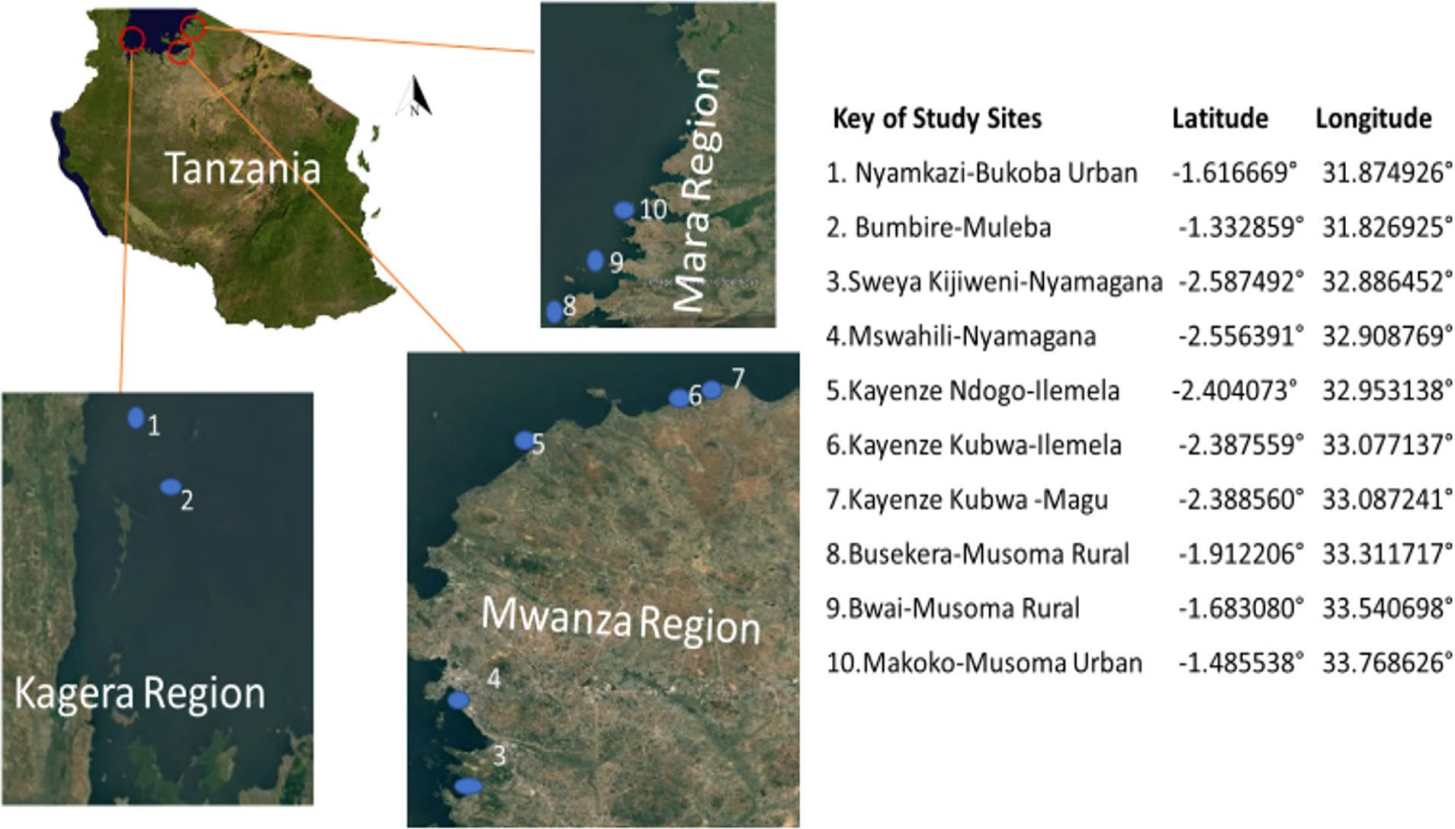
Map showing the geographical locations of the study sites along the Lake Victoria basin in northwestern Tanzania. The sites are distributed across three regions: Kagera, Mwanza, and Mara. Blue dots (1–10) represent specific sampling points selected for the study, including areas in Muleba, Bukoba, Nyamagana, Ilemela, Magu, and Musoma districts. Coordinates (latitude and longitude) of each site are detailed in the accompanying key to facilitate spatial referencing.

A cross‐sectional study was conducted in the Lake Victoria basin, covering districts in Mwanza (Nyamagana, Ilemela, Magu), Kagera (Muleba, Bukoba urban), and Mara (Musoma urban, Musoma rural) regions of Tanzania, because of the vast sardine landing stations. Lake Victoria covers 35,088 km^2^, shared by Tanzania (51%), Uganda (43%), and Kenya (6%). It extends 412 km north to south and 355 km west to east, with a capacity of 2760 km^3^, a depth of 1134 m above sea level, an average depth of 40 m, and a maximum depth of 80 m (Figure 1).

### 2.2 Selection of Study Participants, Interviews and Sample Collection

With support from the beach management unit, study participants were selected from sardine processing stations in representative districts in Mwanza, Kagera, and Mara regions using a simple random sampling technique. Each male and female adult between the ages of 18 and 68 involved in sardine processing at the time of this study had an equal chance of being selected. The number of study participants was estimated using Yamane's formula (Yamane 1967) to obtain 425 respondents for the assessment of awareness of heavy metal contamination and sardine handling practices at a 95% confidence interval.

A semi‐structured questionnaire in Kobotoolbox software was used to capture demographic characteristics (occupation, gender, age, marital status, income level and education), information on sardine consumption frequency, awareness of heavy metal content, and their contamination in sardine. A total of 279 sardine packed samples from Mwanza, Kagera, and Mara were collected for laboratory analysis. The distribution of sample size from each region varied depending on the available sardine landing stations, giving Mwanza (114), Kagera (78) and Mara (87). Samples were taken from every quadrat of the spread‐drying sardine or from the bottom, middle, and top of the sack to get aggregate samples. The aggregate was thoroughly mixed, and about 0.5 kg of sample was obtained. Additionally, 0.5 kg of fresh sardines were sampled directly from the cannons at the landing stations. To avoid cross‐contamination, samples were packed in individual polyethylene bags, clearly labeled, and placed in Styrofoam boxes, and transported to the National Fish Quality Control Laboratory (NFQCLAB) for heavy metal analysis. Samples of fresh sardine were stored in separate boxes with temperatures maintained at about 4°C using ice cubes placed inside the Styrofoam boxes to maintain freshness. Upon arrival at the laboratory, fresh samples were stored in a sample fridge at 1°C to 4°C, while dried samples were kept at room temperature on designated sample racks.

### 2.3 Determination of Heavy Metals in Sardine

About 0.5 kg of fresh and dried sardine samples were homogenized using a heavy‐duty laboratory blender. Two (2) g of the homogenized samples were weighed into microwave digestor tubes. A high purity nitric acid 7.5 mL (Sigma Aldrich,70%) and 7.5 mL of hydrogen peroxide (Labtech, 30%) were added to each tube. The tubes were then placed in the microwave digestor (Milestone, Ethos Easy 20,044,924) for an hour to complete digestion. Digestion in the microwave was performed at a controlled temperature program: heating to 180°C for 20 min, holding for 15 min at 180°C, and cooling to 33°C for 35 min. Microwave sample digestion was opted due to short digestion time, less acid consumption, and high extraction efficiency (Güven and Akinci 2011). The digested samples were then filtered using Whatman filter paper (No. 42) and diluted with deionized water to a final volume of 50 mL. Acid blanks (laboratory blanks) were made at each stage of the digestion to ensure the chemicals were not contaminated. Each set of digestion had its own acid blank that was also used for correction. To ensure that the equipment read only the precise values of heavy metals, blank samples were tested by Flame Atomic Absorption Spectrophotometry FAAS (AAS 280 FS Varian Australia PTY LTD) before the samples, and their values were subtracted to obtain the actual sample concentration. The FAAS was operated at air‐acetylene flame type, 5 mA lamp current, and 0.5 nm slit width for both test heavy metals, and 228.8 and 324.7 nm wavelength for Cd and Pb respectively.

The results were expressed in mg of heavy metal/kg (dry and wet weight) of sardine (EPA 2007).

$$\text{Metal concentration}\frac{\mathrm{mg}}{\mathrm{kg}}=\frac{\text{Reading of atomic absorption}\;\left(\mathrm{mg}/L\right)\times \text{Volume of diluent}\;\left(\mathrm{mL}\right)}{\text{Sardine sample weight}\;\left(g\right)}$$

Fresh samples were analyzed directly without further drying. Thus, to ensure accuracy and reliability in the findings, the concentration of heavy metals in fresh sardine samples was adjusted for a moisture content of 68.0%, as determined by the method (Manahan 2022). Then the adjusted concentration of heavy metal on a dry weight basis was calculated as per the formula below.

$$\text{Metal concentration}\frac{\mathrm{mg}}{\mathrm{kg}\left(\mathrm{dry}\;\text{weight}\right)}=\frac{\;\text{Concentration}\;\mathrm{mg}/\mathrm{kg}\left(\mathrm{wet}\;\text{weight}\right)}{1-\%\text{Moisture content}}$$

To ensure quality control, all glassware and sample vials were properly washed with detergent, soaked in 10% nitric acid for an hour followed by a thorough rinse with deionized water, then dried in an oven. All reagents were high‐quality analytical grades. The instrument was calibrated, and up to five known concentrations of cadmium and lead standards (Sigma Aldrich) were used to obtain a calibration curve. The FAAS was re‐calibrated after every twenty runs. The digestion mixture without the samples was used as a blank and analyzed. The recovery of the spiked samples was calculated to determine the performance of an analytical technique. Recoveries of heavy metals from spiked samples were 93.5% for Cd and 102% for Pb, which were within the acceptable range (Onyele and Anyanwu 2018). Linearity (*R* ^2^) from calibration standards was 1.0, implying perfect linearity. Recoveries were calculated as shown below (Wang et al. 2020).

$$\text{Recovery}\;\left(\%\right)=\frac{\text{Obtained concetration}\;\left(\mathrm{mg}/\mathrm{kg}\right)}{\text{Spiked concentration}\;\left(\mathrm{mg}/\mathrm{kg}\right)}x100$$

### 2.4 Human Health Risk Assessment

Likelihood of noncarcinogenic and carcinogenic risks associated with exposure to heavy metal due to consumption of sardine was also assessed. Estimated Daily Intake (EDI) of heavy metals was computed based on the concentrations of these metals in sardine (C) and the average daily consumption of sardine in grams (W) per kilogram of Tanzanian body weight (BW) for adults, both male (62.8 kg) and female (57.7 kg).

$$\mathrm{EDI}=\frac{C\times W}{\mathrm{BW}}$$

Target Hazard Quotient (THQ) for noncarcinogenic risk was then calculated by comparing the EDI to the oral reference dose (RfD) of Cd (1.0 × 10^−3^ mg/kg/day) and Pb (4.0 × 10^−3^ mg/kg/day) (EPA 1998, 2000), representing the level of exposure considered safe over an extended period.

$$\mathrm{THQ}=\frac{\mathrm{EDI}}{R\mathrm{fD}}$$

Hazard Index (HI), indicating cumulative noncarcinogenic effects, was derived as the sum of THQs for Cd and Pb (EPA 2001). HI exceeding 1.0 indicates potential health risks (EPA 2000).

$$\mathrm{HI}=\mathrm{THQ}\left(\mathrm{Cd}\right)+\mathrm{THQ}\left(\mathrm{Pb}\right)$$

Conversely, the Target Cancer Risk (TR) for carcinogenic risk was determined by multiplying the EDI by the respective cancer oral slope factor (CSF) for Cd (6.3) mg/person/day and Pb (8.5) mg/person/day, representing the probability of cancer development in the long term (EPA 2000; OEHHA 2009; as cited in Akter Laboni et al. 2022).

TR=EDI×CSF

TR < 1 × 10^−6^ imply no public health concern, TR > 1 × 10^−4^ is not acceptable and when TR lies between 1 × 10^−6^ and 1 × 10^−4^, it calls for attention to reduce the risk (EPA 2001).

### 2.5 Data Processing and Analysis

Data were imported into Stata Version 17 for analysis. The Levene's test was performed to check the homogeneity of variances of heavy metal concentrations in the sardine across variables of interes. The assumption of homogeneity of variances was violated. Due to the violation of the homogeneity of variances assumption, analysis of variance (ANOVA) was performed using a robust method (Kruskal‐Wallis nonparametric). Median concentrations of Cd and Pb were reported with standard deviations (SD), and statistical significance was determined at *p* < 0.05. The calculation of the risk for noncarcinogenic and carcinogenic effects, including EDI, THQ, HI, and TR respectively, was done in Microsoft Excel 2019.

## 3 Results

### 3.1 Socio‐Demographic Characteristics of the Study Population

A total of 425 participants were enrolled in the study, out of which 80.2% were women (Table 1). Only 12.3% of participants were aged 18 to 29, while the majority (67.5%) were between 30 and 44 years. About 6.1% of the participants were single, and the majority (74.4%) were married. About 85.8% of participants had at most a primary level of education, with only 14.2% having secondary or higher education. Sardine processing was the main occupation accounting for 98.4% of the participants, while fishing was practiced by only 0.5%.

**TABLE 1:** Socio‐demographic characteristics of study participants.

Characteristics	Percent (%)	Number (*n*)
Sex		
Male	19.8	84
Female	80.2	341
Age categories (years)		
18–29	12.3	52
30–44	67.5	285
45+	20.1	85
Marital status		
Divorced	12.5	53
Married	74.4	316
Single	6.1	26
Widowed	7.1	30
Education level		
No formal education	1.2	5
Primary education	84.6	359
College/University education	14.2	60
Family size groups		
< 6	59.2	250
6+	40.8	172
Income categories		
< 5000	7.3	29
5000‐10,000	48.6	193
10,000+	44.1	175
Main occupation		
Fishing	0.5	2
Sardine processing	98.4	418
Sardine trading	1.6	7

### 3.2 Community Awareness of Heavy Metal Contamination of Sardine

Of 425 participants, 96.2% were unaware of heavy metal contamination. Among 16 who knew of heavy metals, 9 identified mercury (77.8%). Only 12 associated drying sardines on bare sand with contamination. About 7 (43.8%) identified sources like mining and industry, with 81.3% learning from media. Cancer was the health concern mentioned most (43.8%) in Table 2.

**TABLE 2:** Community awareness of heavy metals, their sources, health implications, and exposure routes through sardine handling (*n* = 424). Data are presented as percentages (%) and frequencies (*n*).

Awareness level	Percentage (%)	Number (*n*)
Heard of heavy metals		
Yes	3.8	16
No	96.2	408
Types of heavy metals		
Mercury	1.7	7
Lead	0.2	1
Cadmium	0.2	1
Sources of heavy metals contamination		
Mining	1.7	7
Industry	1.7	7
Sand‐drying can expose sardine to heavy metals		
Yes	2.8	12
No	0.9	4
Health implications of heavy metals		
Miscarriage	0.2	1
Kidney failure	0.2	1
Diarrhea	0.2	1
Death	0.5	2
Source of information on heavy metals		
Expert talk	0.2	1
School	0.5	2
Media (television, radio)	3.1	13

### 3.3 Handling Practices of Sardine

In Mwanza, Mara, and Kagera around Lake Victoria, the majority reported using the sun‐drying (97.6%) processing method, with sun‐drying on fishing nets (buti) being the most *common* drying practice (56.2%) and few on bare sand (9.0%) in Table 3.

**TABLE 3:** Sardine handling practices among sardine processors (*n* = 421).

Handling practices	Percent (%)	Number (*n*)
Processing methods		
Sun‐dried	97.6	411
Deep‐fried	2.4	10
Sun‐drying practices		
Rack	27.5	113
Buti	56.2	231
Rock	7.3	30
Sand	9.0	37
Reasons for drying on rack, buti, sand, or rock		
Not contacting sand for human health	55.5	236
Consumers' preferences	27.3	116
Others	17.2	73
Storage/packaging		
Sack	98.6	413
Box	1.4	6

### 3.4 Heavy Metals Contamination of Sardine

A total of 279 sardine samples were analyzed for cadmium and lead, with none exceeding the 0.3 mg/kg limit FAO/WHO (1972). The mean cadmium concentration was 0.012 ± 0.005 mg/kg, with sand and rack sun‐dried sardines recording the highest levels. The mean lead concentration was 0.081 ± 0.057 mg/kg (Table 4). Spatial variations in heavy metals were observed, with Nyamagana in Mwanza having higher lead levels compared to Magu. Prior to adjusting for the water content of fresh sardine, the concentration of heavy metals in dried sardine was slightly higher, about 1.3 times, than in fresh sardine. However, after adjustment, the concentration increased substantially to approximately 3 times higher.

**TABLE 4:** Concentration of Cd and Pb (mg/kg) in sardine samples from Lake Victoria.

	*N* (%)	Cd (mg/kg)					Pb (mg/kg)				
		Mean	SD	Median	*p* ^1^	*p* ^2^	Mean	SD	Median	*p* ^1^	*p* ^2^
**Region**											
Kagera	77 (27.8)	0.012	0.005	0.012	0.1756	0.1745	0.08	0.055	0.063	0.5586	
Mara	86 (31.1)	0.012	0.005	0.012			0.082	0.053	0.088		
Mwanza	114 (41.2)	0.013	0.006	0.012			0.083	0.062	0.079		
**Fish State**											
Dried	210 (75.8)	0.013	0.006	0.014			0.096	0.054	0.097		
Fresh	67 (24.2)	0.01	0.003	0.01	0.0001		0.038	0.044	0.065	0.000	
Fresh^†^	67 (24.2)	0.031	0.009	0.031	0.0001		0.119	0.138	0.203	0.001	
**Fish processing methods**											
Sun‐dried	197 (93.8)	0.013	0.006	0.013			0.013	0.005	0.013		
Fried	13 (6.2)	0.004	0.002	0.004	0.0001	0.000	0.092	0.02	0.085	0.0001	0.000
**Sun‐drying practices**											
Buti	87 (44.2)	0.012	0.005	0.013			0.098	0.053	0.1		
Rack	81 (41.1)	0.016	0.005	0.017	0.0001	**0.000**	0.083	0.048	0.092	0.0004	**0.032**
Rock	10 (5.1)	0.01	0.005	0.01			0.151	0.026	0.142		
Sand	19 (9.6)	0.016	0.005	0.017			0.116	0.093	0.09		
**Districts**											
*Kagera*											
Bukoba	29 (37.7)	0.012	0.004	0.011			0.092	0.06	0.084		
Muleba	48 (62.3)	0.012	0.005	0.013	0.6164		0.073	0.051	0.056	0.2742	
*Mara*											
Musoma district	48 (62.3)	0.013	0.005	0.013	0.0001		0.093	0.045	0.1		
Musoma urban	28 (33.3)	0.008	0.004	0.007			0.058	0.062	0.065	0.009	
*Mwanza*											
Ilemela	63 (55.3)	0.014	0.007	0.017		**0.0005**	0.082	0.039	0.085		
Magu	26 (22.8)	0.013	0.004	0.011			0.079	0.099	0.057		
Nyamagana	25 (21.9)	0.009	0.004	0.01			0.089	0.061	0.09		0.3201
**Total**	**277**	**0.012**	**0.005**	**0.012**			**0.082**	**0.057**	**0.081**		

*Note:* Bold indicates statistical significance. *N* (%) indicates the number and proportion of samples. *p* ^1^ is from the Kruskal–Wallis test (nonparametric, based on median), and *p* ^2^ is from one‐way ANOVA (parametric, based on mean where assumptions were met).

^†^ Is the concentration of Cd and Pb after correction for moisture content; SD is standard deviation.

### 3.5 Health Risk Assessment

The health risk of cadmium and lead due to consumption of sardines was estimated and presented as Estimated Daily Intake (EDI), Target Hazard Quotient (THQ), Hazard Index (HI) and Target Cancer Risk (TR). EDI values for Cd ranged from 1.043 × 10^−5^ to 1.175 × 10^−5^ mg/kg/day, and for Pb from 2.704 × 10^−6^ to 3.047 × 10^−6^ mg/kg/day. THQ values for Cd ranged from 0.0027 to 0.0031, and for Pb from 0.0026 to 0.0029. HI values ranged from 0.0053 to 0.0060. TR values for Cd ranged from 1.70 × 10^−5^ to 1.92 × 10^−5^, and for Pb from 1.03 × 10^−9^ to 9.99 × 10^−5^ as per Table 5.

**TABLE 5:** Estimated daily intake (EDI), target hazard quotient (THQ), hazard index (HI), and target cancer risk (TR) of cadmium (Cd) and lead (Pb) from sardine consumption among adult male and female consumers in Mwanza, Kagera, and Mara regions of Tanzania.

Heavy metals	Mwanza		Kagera		Mara	
	Male	Female	Male	Female	Male	Female
**Estimated daily intake (EDI) in mg/kg/day**						
Cd	1.165 × 10^−5^	1.175 × 10^−5^	1.043 × 10^−5^	1.161 × 10^−5^	1.1676 × 10^−5^	1.134 × 10^−5^
Pb	3.019 × 10^−6^	3.047 × 10^−6^	2.704 × 10^−6^	2.959 × 10^−6^	3.026 × 10^−6^	2.941 × 10^−6^
**Target hazard quotient (THQ) and Hazard Index (HI)**						
THQ (Cd)	0.0030	0.0031	0.0027	0.0027	0.0030	0.0029
THQ (Pb)	0.0029	0.0029	0.0026	0.0029	0.0029	0.0028
HI	0.0059	0.0060	0.0053	0.0056	0.0059	0.0058
**Target cancer risk (TR)**						
Cd	1.9 × 10^−5^	1.92 × 10^−5^	1.7 × 10^−5^	1.70 × 10^−5^	1.7 × 10^−5^	1.85 × 10^−5^
Pb	9.9 × 10^−5^	9.99 × 10^−5^	1.03 × 10^−9^	9.87 × 10^−5^	9.92 × 10^−5^	9.64 × 10^−5^

## 4 Discussion

Over 80% of the study participants who were processing and trading sardine were women, while men were typically involved in fishing. This aligns with findings from the Lake Tanganyika study, where women were observed to significantly dominate the processing of sardine (Gee and Muumin 2023). Few participants were aware of heavy metal contamination in sardine, and among them, some linked the contamination to mining and industrial activities (Table 2).

It was found that the level of education was the most important determinant of participants' awareness of heavy metal contamination. Specifically, those who were aware had secondary or higher levels of education, while none with primary education were aware of heavy metal contamination. A related study reported a direct correlation between the level of education and awareness of food contamination (Ssanyu et al. 2023).

Of those who were aware of heavy metals, the majority mentioned mercury as the type of heavy metal they have heard most. Some mentioned health implications from heavy metals such as vomiting, diarrhea, miscarriage, kidney failure, cancer, and death. A study in Uganda also found cancer and kidney dysfunction associated with heavy metals contamination awareness (Ssanyu et al. 2023). Even though the majority of the participants in the current study were not aware of heavy metal contamination, they had a general understanding of physical contamination. For instance, the majority of the participants reported drying sardines on racks, buti, and rock to avoid contact with sand as it affects human health. The practical implication is that contact between sardines and sand or bare ground may introduce dirt, microorganisms, or fecal matter and chemical contaminants into the fish. While such contaminants lower the quality and market value of sardines, consumption of such contaminants can lead to gastrointestinal illnesses and other health risks. This understanding is supported by findings from a microbiological quality study, which reported that sardines dried on racks were free from *E. coli* and *Salmonella* spp., indicating that such methods help prevent fecal contamination and enhance microbial safety (Baniga et al. 2017). The improvement in sardine handling practices on raised racks and buti was attributed to government interventions aimed at promoting value addition and enhancing the economic potential of sardines for human consumption, particularly for export to Rwanda. For instance, in Muleba district, Kagera region, many respondents reported that enforcement efforts were initiated under the leadership of the late President John Pombe Magufuli through the Fisheries authorities (Ministry of Livestock and Fisheries and local government). These efforts mandated sardine processors to construct raised drying racks to improve hygiene and reduce risks of contamination. Additionally, Rwandan importers required products to be handled in hygienic conditions, which further motivated improvements in postharvest practices. In some parts of Mwanza region, the construction of some improved drying racks was supported under the Tanzania Agricultural Support Project Phase II (TASP II), implemented by the National Fish Quality Control Laboratory (NFQCL).

The majority of participants expressed concerns that sand contaminated with feces or any chemicals could cross‐contaminate sardine once in contact, and when these sardine are consumed by humans, they pose significant health risks such as stomach pain and/or diarrhea. They further reported that the presence of sand in sardines could increase the risk of appendicitis due to the accumulation of sand in the appendix. However, health education campaigns related to fish safety and handling remain insufficient, with limited funding and inadequate contextualization to local practices and knowledge systems. Inadequate coordination among departments could further weaken outreach, leaving communities unaware of safe fish handling practices. This was reflected in survey and interview responses, where many fish processors reported having received little to no formal information from extension services about heavy metals contamination and associated risks (Table 2).

The majority of those who were aware of heavy metals reported that drying sardine on sand could expose them to heavy metal contamination, contrary to the belief that sardine were dried on sand for quick drying, which was acceptable regardless of the quality and safety concerns (Kabahenda et al. 2009).

Although sun‐drying is a common method for drying sardines, traditional practices such as drying fish directly on bare ground, sand, or uncovered surfaces near roads, polluted water bodies, or industrial areas can increase the risk of contamination from dust, soil, and airborne pollutants. However, in the present study, relatively low concentrations of contaminants were observed. This may be attributed to improved drying practices adopted by the sardine processors, including the use of drying racks (44%) and buti (41%), which provide better protection from environmental contaminants compared to sand drying (9.6%) (Table 3). These improved methods help minimize contact with contaminated surfaces and reduce exposure to pollutants, thereby enhancing the safety of dried fish. This highlights the need to consider drying conditions when interpreting heavy metal levels in sun‐dried fish.

The overall median concentrations of cadmium (Cd) and lead (Pb) in sardines were within the tolerable limits established by the Food and Agriculture Organization (FAO 1989). The low concentration of heavy metals could be attributed to the improved sardine drying practices, specifically the use of buti and racks, which prevent contact with sand and other contaminants including heavy metals. These findings align with other studies. A comparative study by Thomas (2016) on sun‐dried sardines from offshore Mwanza reported a low Pb concentration of 0.01 mg/kg. However, the study did not provide a detailed methodology regarding the specific landing sites or the drying methods used, for example, drying on bare ground, racks, or rocks, which are important factors that could influence contamination levels. A study in Egypt reported low levels of Cd (0.14 mg/kg) and Pb (0.16 mg/kg) in sardines (Monier et al. 2023) and Cd (0.0044 to 0.1 mg/kg) and Pb (0.011 to 0.27 mg/kg) in other fish species in Brazil (Tarley et al. 2001). Conversely, a related study from the Kenyan side of Lake Victoria has reported higher concentrations of Cd (2.5 ± 0.13 mg/kg) and Pb (3.5 ± 0.41 mg/kg) in Tilapia and Nile perch respectively (Foo et al. 2021). Also, a study in Uganda reported cadmium (Cd) concentrations of 0.57 μg/g in sun‐dried *Rastrineobola argentea* collected from 10 landing sites on Lake Victoria (Mbabazi and Wasswa 2010).

Despite heavy metals in dried and fresh sardines being within tolerable limits, significant variation in concentration was observed signifying the influence of water content on the concentration of heavy metals in sardine. Before adjusting for water content, dried sardine had slightly higher heavy metal concentration at about 1.3 times that of fresh sardine. After adjustment, the concentration was approximately 3 times higher. This argument is supported by the study's methodology, where fresh sardine samples were analyzed without drying to allow comparability for true heavy metal levels in the solid tissue. This highlights the importance of adjusting water content to prevent dilution effects and ensure accurate reporting, as heavy metals primarily bind to the solid tissue portion, leading to significantly higher concentrations on a dry weight basis.

Globally, the presence of cadmium (Cd) and lead (Pb) in sardines is an increasing cause for concern due to their potential health risks. Some countries reported higher concentrations; for example, in Algeria, sardine ( *Sardina pilchardus* ) had Cd and Pb levels of 0.62 and 0.55 mg/kg, respectively (Mehouel et al. 2019), while in Turkey, Cd and Pb levels in fresh sardines were 0.46 mg/kg. Other studies reported low concentrations of Cd (0.1 mg/kg) and Pb (0.3 mg/kg) in both fresh and dried fish, including *Epinephelus areolatus* and *Sardinella longiceps* in India (Arisekar et al. 2023) and Cd (0.62–1.37 mg/kg) and Pb (0.39–4.51 mg/kg) in Colombia (Alcala‐Orozco et al. 2021). These variations may be due to differences in environmental pollution, fish handling, and processing methods.

In the current study, a higher concentration of heavy metals in dried compared to fresh sardine might be due to the larger sample size of dried sardine (75.8%) compared to fresh (24.2%). This was attributed to the inclusion of various drying practices such as rock, sand, rack, and buti compared to the fresh samples (Table 3). A large sample size often transforms small changes into statistically significant variations (Faber and Fonseca 2014). Additionally, mean concentrations across drying methods differed significantly (*p* < 0.05). Sun‐dried sardines had the highest median Cd concentration compared to fried sardines, likely due to the larger sample size of sun‐dried sardines (93.8%) compared to fried sardines (6.2%) as per Table 4. The larger rack sample resulted from improved drying practices using racks instead of sand, driven by consumer demand and government intervention.

Similarly, heavy metal contamination varied across districts within the region. In Mwanza, Ilemela had the highest median Cd concentration compared to Nyamagana and Magu, likely due to factors such as cross‐contamination during drying, differences in metal accumulation, and Ilemela's larger sample size (55.3%) compared to Nyamagana (21.9%) and Magu (22.8%) in Table 4. Furthermore, localized sources of contamination such as industrial runoff, poor waste disposal, agricultural activities, and proximity to urban developments may have contributed to the elevated concentrations observed in Ilemela. A previous study by Mbabazi and Wasswa (2010) reported that higher levels of heavy metals in sun‐dried sardine samples from ten landing sites around Lake Victoria in Uganda were likely due to untreated industrial discharges and other human‐related activities. This study concludes that heavy metal contamination could be attributed largely to anthropogenic activities. While the present study focused on sardine alone, studies in Tanzania have reported heavy metal contamination of sediments and water, providing useful information to support our findings. A study by Mhina (2016) reported low levels of Cd (1.61 × 10^−2^ to 3.91 × 10^−2^ mg/kg) and Pb (5.20 × 10^−1^ to 1.09 mg/kg) in sediments, and Cd (2.30 × 10^−3^ to 3.11 × 10^−2^ mg/L) and Pb (1.30 × 10^−4^ to 9.95 × 10^−2^ mg/L) in water at designated sampling sites in Lake Victoria. Though this study is about a decade and a half apart from our study, the low levels of Cd and Pb in sediment and water may imply the current low levels in sardine.

The EDI values for Cd and Pb in all regions, for both males and females, were below the FAO/WHO provisional tolerable daily intake (PTDI) for Cd (8.0 × 10^−3^ mg/kg/day) and Pb (5.0 × 10^−2^ mg/kg/day). This implies that the concentrations of these metals in sardines were within acceptable limits for lifetime consumption without significant health risks (FAO/WHO 1972). Studies in other countries also have reported low EDI for heavy metals in fish; in Ghana, Cd (99.99 × 10^−6^ mg/kg/day) and Pb (5.31 × 10^−6^ mg/kg/day) (Kwaansa‐Ansah et al. 2018) and in Egypt, Cd and Pb (0.006 and 0.016 mg/kg/day) in fish species including sardine (Alcala‐Orozco et al. 2021).

The THQ values for the metals studied in sardines were below one, indicating no significant noncarcinogenic health risks for consumers (FAO 1989). There was no significant variation of THQ between males and females in all regions. This could be due to the fishing communities of Lake Victoria, where fish is a staple food, and the dietary habits between males and females are often culturally and economically driven, leading to similar consumption patterns. Similar study in Tanzania reported low THQ of 0.113 and 0.189 for Cd and Pb in Nile perch from Lake Victoria (Mhina 2016). Comparative studies in Thailand and Bangladesh also recorded low THQ for Cd and Pb in fish species (Tanhan et al. 2023; Alam et al. 2023).

The HI values for Cd and Pb were below one for both males and females, indicating no significant non‐carcinogenic health risks (EPA 2000; Onyele and Anyanwu 2018). The study on Nile perch from Lake Victoria in Tanzania also reported a low HI (0.302) (Mhina 2016).

The TR values for all metals assessed in sardines were below 1.0 × 10^−4^, indicating no significant carcinogenic health impact. Similarly, other studies reported low TR values for Cd and Pb in various fish species, ranging from 1.68 × 10^−10^ to 6.75 × 10^−5^ (Tanhan et al. 2023; Alam et al. 2023).

Although Cd and Pb levels in the current study were within permissible limits, these metals are highly toxic even at low concentrations and are listed among the top ten chemicals of major public health concern globally (WHO 2010). Long‐term exposure through food (e.g., contaminated fish) or the environment can result in cumulative toxic health effects after years or decades (Järup 2003). Lead can affect the brain and nervous system, leading to learning and memory problems in children (Fatima et al. 2024; Tiwari et al. 2024). Both lead and cadmium can raise the risk of heart problems such as high blood pressure, irregular heartbeat, and hardening of the arteries due to their effects on body cells (Fatima et al. 2024; Pan et al. 2025; Kuo et al. 2024). A study from Poland also showed that fewer heart‐related deaths occurred when air levels of these metals were lower (Marchwińska‐Wyrwał et al. 2010). These metals can also damage the kidneys, and cadmium has been linked to cancers of the lung, bladder, and liver (Fatima et al. 2024; Tiwari et al. 2024). They may weaken the immune system by reducing the function of important immune cells, making the body more likely to infections and allergies (Skoczyńska et al. 2002).

Overall, this study highlights the community awareness of heavy metals and provides scientific evidence of heavy metal contamination of sardine, estimated dietary exposure to humans, and potential health risks. Information obtained from this study has the potential to inform awareness campaigns to the public, including traders and consumers, and guide actions and decisions among relevant sectors such as environmental, food regulatory authorities, and the fishing community.

Nevertheless, the study presents some limitations, including a higher representation of women in the occupation, affecting the estimation of daily intake of heavy metals as consumption varies by sex. The risk assessment focused on adults, excluding children, despite age‐related susceptibility to chemical intake and exposure. The study was conducted at the end of the wet and the beginning of the dry season between May and July, lacking seasonal representation and potentially leading to an underestimation or overestimation of heavy metals in sardines. Also, the study did not analyze heavy metals in water and sediment that would have given room for further triangulation of the results. Due to the lack of data on cadmium, the sample size calculation relied on lead prevalence data to represent both. The study did not measure the actual weight of participants but rather used the average Tanzanian weights for males and females to calculate risk.

Considering these limitations, several methodological considerations may improve the accuracy and robustness of future dietary risk assessments: Balanced recruitment of male and female participants at the household level may better reflect gender‐specific consumption patterns. Inclusion of vulnerable groups, such as children and the elderly, is important, as their developing or declining physiological systems, metabolic rates, and dietary habits may increase their susceptibility to the adverse effects of toxicants. The use of measured body weights, rather than national averages, could enhance the precision of intake estimates. Accounting for seasonal variation would help capture potential fluctuations in heavy metals levels across different times of the year. Furthermore, concurrent analysis of heavy metals in fish, water, and sediment would support triangulation of exposure sources and strengthen interpretation of contamination pathways.

## 5 Conclusions and Recommendations

The study assessed community awareness of heavy metals, sardine handling practices, and the risk of dietary exposure to metals due to consumption of sardine from Lake Victoria, Tanzania. While awareness of heavy metals and their contamination of sardine was generally low, participants recognized potential health risks of sand as physical contamination. The level of education correlated with awareness of heavy metals. Concentrations of cadmium and lead in sardine were below limits, and the estimated daily intake was within acceptable levels. Hazard quotients and cancer risk were below critical limits, indicating no significant noncarcinogenic and carcinogenic health risks. Based on the findings of this study, sardine from Lake Victoria is considered safe for human consumption. However, local authorities and food safety agencies should promote safer fish drying methods, such as the adoption of raised drying racks, to mitigate contamination risks from soil, dust, and other environmental sources. Regular community education and capacity‐building initiatives on hygienic fish handling and drying practices are vital for empowering stakeholders to reduce contamination and protect consumer health. Continuous monitoring of heavy metal concentrations in sardines from Lake Victoria is essential to ensure timely changes for intervention.

## Author Contributions

**Happiness Kemilembe Venant:** conceptualization (equal), data curation (equal), formal analysis (lead), investigation (lead), methodology (equal), validation (lead), visualization (equal), writing – original draft (equal), writing – review and editing (equal). **Dickson W. Lwetoijera:** conceptualization (equal), data curation (equal), formal analysis (supporting), investigation (supporting), methodology (equal), supervision (supporting), validation (equal), visualization (equal), writing – original draft (equal), writing – review and editing (equal). **Neema Kassim:** conceptualization (equal), data curation (equal), formal analysis (equal), investigation (equal), methodology (equal), supervision (lead), validation (equal), visualization (equal), writing – original draft (equal), writing – review and editing (equal).

## Data Availability

The data that support the findings of this study are available from the corresponding author upon reasonable request.
